# *tomoCAM*: fast model-based iterative reconstruction via GPU acceleration and non-uniform fast Fourier transforms

**DOI:** 10.1107/S1600577523008962

**Published:** 2024-01-01

**Authors:** Dinesh Kumar, Dilworth Y. Parkinson, Jeffrey J. Donatelli

**Affiliations:** a Mathematics Department, Lawrence Berkeley National Laboratory, Berkeley, CA, USA; b Center for Advanced Mathematics for Energy Research Applications, Lawrence Berkeley National Laboratory, Berkeley, CA, USA; c Advanced Light Source, Lawrence Berkeley National Laboratory, Berkeley, CA, USA; Tohoku University, Japan

**Keywords:** X-ray tomography, micro-CT, synchrotron tomography, GPU, MBIR, nano-CT, tomographic reconstruction

## Abstract

X-ray-based computed tomography is a well established technique for determining the three-dimensional structure of an object from its two-dimensional projections. In the past few decades, there have been significant advancements in the brightness and detector technology of tomography instruments at synchrotron sources. These advancements have led to the emergence of new observations and discoveries, with improved capabilities such as faster frame rates, larger fields of view, higher resolution and higher dimensionality. These advancements have enabled the material science community to expand the scope of tomographic measurements towards increasingly *in situ* and *in operando* measurements. In these new experiments, samples can be rapidly evolving, have complex geometries and restrictions on the field of view, limiting the number of projections that can be collected. In such cases, standard filtered back-projection often results in poor quality reconstructions. Iterative reconstruction algorithms, such as model-based iterative reconstructions (MBIR), have demonstrated considerable success in producing high-quality reconstructions under such restrictions, but typically require high-performance computing resources with hundreds of compute nodes to solve the problem in a reasonable time. Here, *tomoCAM*, is introduced, a new GPU-accelerated implementation of model-based iterative reconstruction that leverages non-uniform fast Fourier transforms to efficiently compute Radon and back-projection operators and asynchronous memory transfers to maximize the throughput to the GPU memory. The resulting code is significantly faster than traditional MBIR codes and delivers the reconstructive improvement offered by MBIR with affordable computing time and resources. *tomoCAM* has a Python front-end, allowing access from *Jupyter*-based frameworks, providing straightforward integration into existing workflows at synchrotron facilities.

## Abstract 2

*tomoCAM* is an open-source implementation of model-based iterative reconstructions that reformulates the iterative tomographic reconstruction problem to leverage the faster time complexity of non-uniform fast Fourier transforms. Moreover, it harnesses the computational capabilities of modern GPUs, leading to significant performance gains compared with currently available publicly accessible tools. *tomoCAM* is accessible via Python and is compatible with *NumPy*, facilitating its incorporation into pre-existing workflows at synchrotron light sources.

## 1. Introduction

Micro- and nano-tomography using synchrotron technology is crucial in uncovering the inner makeup of modern materials, particularly in dynamic settings. Its diverse applications include: the investigation of the fractures and deterioration of ceramic matrix composites, which are novel lightweight materials used in jet engines that operate under high temperatures and pressure (Forna-Kreutzer *et al.*, 2021 ▸); the study of the flow of oil, brine and carbon dioxide through rocks (Walsh *et al.*, 2014 ▸); and the analysis of dendrite formation in batteries, which causes capacity reduction and eventual failure (Dienemann *et al.*, 2023 ▸). Many synchrotron micro-computed tomography (micro-CT) facilities now have cameras that can acquire many-megapixel images at thousands of frames per second (MacDowell *et al.*, 2012 ▸; Nikitin *et al.*, 2022 ▸; Ge *et al.*, 2018 ▸; Thuering *et al.*, 2011 ▸; Mokso *et al.*, 2017 ▸). These advances in instrumentation have encouraged users to push the boundaries of what can be imaged at synchrotron beamlines.

An increasing number of investigators are conducting *in situ* (Larson & Zok, 2018 ▸; French *et al.*, 2022 ▸) and *in operando* (Kulkarni *et al.*, 2020 ▸; Dienemann *et al.*, 2023 ▸) measurements. Typically the initial technique attempted for micro-CT reconstructions is filtered back-projection (FBP)^1^, which is available in *tomopy* (Gürsoy *et al.*, 2014 ▸), *tomocupy* (Nikitin, 2023 ▸), *ASTRA* (van Aarle *et al.*, 2015 ▸, 2016 ▸) and *TIGRE* (Biguri *et al.*, 2016 ▸, 2020 ▸). However, in the case of many dynamic experiments, where the specimen under observation is changing rapidly, it is generally not possible to capture sufficient projections to satisfy angular Shannon sampling conditions (Crowther *et al.*, 1970 ▸) and overcome the noise. FBP is not a suitable option in such situations. The reconstructions obtained through this method tend to have excessive noise levels and exhibit streaking artifacts, making it difficult or even impossible to carry out further analysis.

As an alternative, iterative methods, such as the simultaneous iterative reconstruction technique (SIRT) (Tarantola & Valette, 1982 ▸) and model-based iterative reconstruction (MBIR) (Venkatakrishnan *et al.*, 2013 ▸; Mohan *et al.*, 2014 ▸), aim to mitigate these shortcomings. In recent years, significant efforts have been made to develop reconstruction packages that provide access to iterative reconstruction algorithms with total variation constraints. This is primarily driven by the increasing demand for high-quality images in various imaging modalities, such as computed tomography (CT), magnetic resonance imaging and optical microscopy. Some of the prominent efforts include *SVMBIR*, *TIGRE*, *ASTRA*, *ToMoBAR* (Kazantsev & Wadeson, 2020 ▸) and *Core Imaging Library (CIL)* (Jørgensen *et al.*, 2021 ▸). *SVMBIR* is a multi-threaded implementation of MBIR with a Python front-end (SVMBIR, 2020 ▸). *ASTRA* and *TIGRE* provide an array of CPU- and GPU-based algorithms for direct inversion and iterative algorithms with total variation constraints. *ToMoBAR* and *CIL* focus primarily on regularized iterative methods for datasets with sparse projection data.

These iterative algorithms formulate the reconstruction as an optimization problem. The solution is obtained through an iterative process that aims to minimize the mismatch between the measured data and a forward model (Radon transform) of a digital representation of the sample. This iterative approach enables the incorporation of prior knowledge, such as total variation constraints, into the optimization process, as demonstrated in various studies (Trampert & Leveque, 1990 ▸; Zhang *et al.*, 2014 ▸; Venkatakrishnan *et al.*, 2013 ▸; Mohan *et al.*, 2014 ▸). However, current CPU-based implementations of MBIR typically require a large compute cluster to achieve turnaround times that are comparable with data collection times. This not only adds extra time to the experiment-to-analysis loop but also places an additional burden on material scientists, who must acquire a new set of expertise in using a compute cluster. This paper introduces *tomoCAM*, a GPU-accelerated implementation of MBIR that is based on the non-uniform fast Fourier transforms (NUFFT) approach (Greengard & Lee, 2004 ▸; Fessler & Sutton, 2003 ▸; Dutt & Rokhlin, 1993 ▸), which significantly reduces compute time complexity while maintaining accuracy. With the computational power provided by modern GPU devices and the relatively affordable cost of computer memory, it has become possible to perform these reconstructions on a single machine within a reasonable amount oftime.

To design our GPU-accelerated algorithm and implementation, we build Radon and back-projection operators based on NUFFT. We also leverage highly optimized cuFFT libraries that are native to the CUDA software development kit (Vingelmann & Fitzek, 2020 ▸). We follow the mathematical outline laid out by Venkatakrishnan *et al.* (2013 ▸) and Mohan *et al.* (2014 ▸) to add a total variation constraint, which helps in reducing noise while preserving the sharp edges. An important feature of our implementation is the flexibility to introduce a different constraint. The choice of the constraint is not limited by the algorithm design.

We test our computational framework through a series of numerical experiments on known phantoms and experimental data made publicly available through Tomobank (Carlo *et al.*, 2018 ▸). We compare the reconstructions with those obtained from FBP (Gürsoy *et al.*, 2014 ▸) and *SVMBIR* (SVMBIR, 2020 ▸), a publicly available CPU-based package.

In our numerical experiments, we found that: (i) when compared with FBP, the reconstruction quality produced by *tomoCAM* was superior with less noise and required a lower number of projections, and (ii) additionally, we observed that *tomoCAM* was around 15 times faster on a single machine than *SVMBIR* and 22 times faster than *TIGRE* (IRN-TV-CGLS).

Finally, we provide a Python front-end that exposes *tomoCAM* functionality to the widely used *NumPy* package (Harris *et al.*, 2020 ▸), using *pybind11* (Jakob *et al.*, 2017 ▸). This makes it easy to integrate *tomoCAM* into existing workflows. The code is freely available at https://github.com/lbl-camera/tomocam.

## 2. Radon transform and NUFFT

This section provides a brief overview of the fundamental concepts related to tomography, including the Radon transform (as well as its adjoint) and its connection to the Fourier transform. To perform tomographic measurements, a series of images, referred to as projections, are captured at various angles by rotating either the camera or the sample under observation.

### 2.1. Radon transform

**Figure 1:**
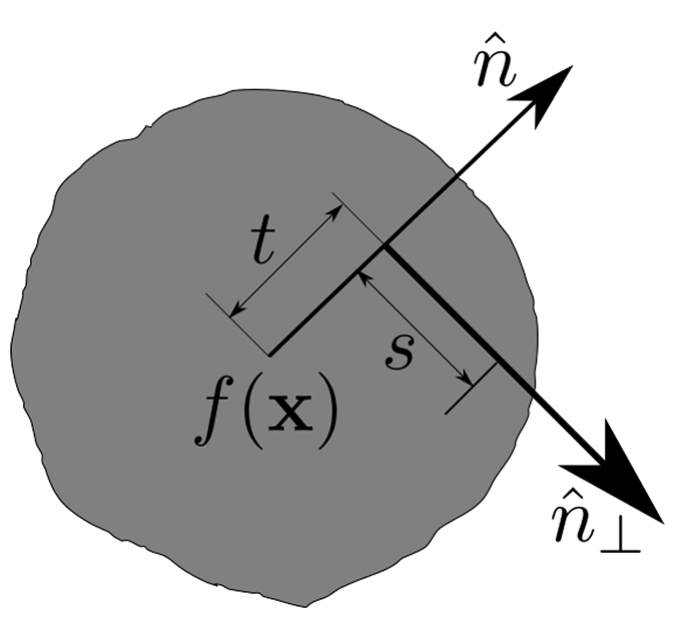
The Radon transform of *f* is its line integral along each line perpendicular to 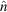 .

The Radon transform is fundamental to any tomographic reconstruction. It transforms a function 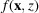 , 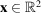 , 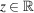 , to 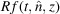 , 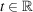 , 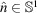 through a line integral (2), see Fig. 1 ▸. Given a set of oriented lines 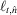 defined as 

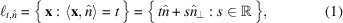

where 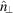 is the direction of the X-ray beam, 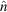 is perpendicular to the beam in same plane as ℓ, and *t* is the distance to ℓ from the origin. The Radon transform *Rf* of function *f* is defined as 

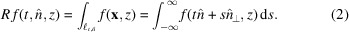

Tomographic measurements can be accurately modeled as the Radon transform of the sample density represented by *f*. It is the inversion of equation (2) that reconstructs *f* from the data, and is of primary importance in tomographic reconstruction. By the central slice theorem, the Fourier transform of *R* *f* in direction 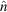 is equivalent to the slice of the Fourier transform of *f* along 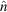 , *i.e.*


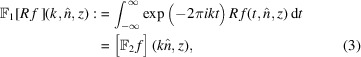

where *z* is the dimension along the axis of rotation and 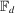 denotes the *d*-dimensional Fourier transform. The Radon transform and its adjoint are two-dimensional operators that are applied slice-by-slice on three-dimensional data. For simplification, we will drop the *z* dependency from the subsequent notations. Assuming *f* and 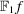 are integrable everywhere, the inverse of the Radon transform (2) is given by

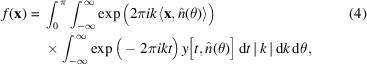

where we denote the θ dependency as 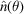 = 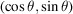 .

It is computationally very expensive to exactly compute equation (4). In practice, (4) is efficiently approximated with a NUFFT (Gürsoy *et al.*, 2014 ▸) or directly estimated in real space through the use of various filters such as Shepp–Logan (Shepp & Logan, 1974 ▸), Ram–Lak (Ramachandran & Lakshminarayanan, 1971 ▸) and Butterworth (Butterworth, 1930 ▸), which approximate and weight by the Fourier sampling density, hence the name ‘filtered back-projection’ (Epstein, 2007 ▸; Jørgensen & Lionheart, 2021 ▸; Candes, 2021 ▸).

These filters are additionally designed to dampen out the higher Fourier frequencies. This is the most commonly used method in the reconstruction of tomographic data, in part because of the sheer speed by which the inversion can be performed. However, in cases when the view is partially blocked, or the specimen is evolving, it may not be possible to collect enough projections to sufficiently sample the Fourier space. In such cases, the FBP results in poor image quality.

### 2.2. NUFFT

On a discrete uniform grid, if a sufficient number of projections are available, inversion of the Radon transform entails computing Fourier coefficients along radial lines using a one-dimensional fast Fourier transform (FFT), followed by two-dimensional backward Fourier transforms from a non-uniform polar grid 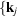 = 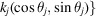 onto a Cartesian grid {(*x*_*n*_, *y*_*n*_)}, which can be represented as the summation 

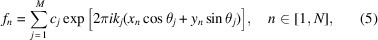

where *c*_*j*_ is the Fourier coefficient at **k**_*j*_, *N* is the number of discrete points that represent the sample density *f* on a uniform Cartesian grid, and *M* is the number of polar grid points representing the projection data. However, directly computing equation (5) is computationally expensive, as the complexity is 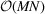 . Data taken at synchrotron light sources can usually reach up to *M* = 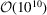 pixels, and the final reconstructed image size *N* has a similar order or magnitude for the final reconstructed image.

**Figure 2:**
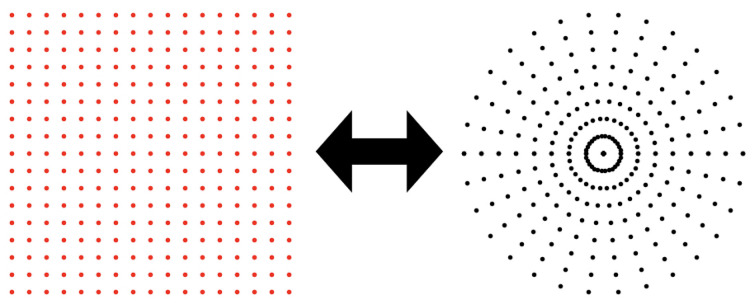
The NUFFT is used to transform intensity on a uniform grid to its Fourier transform on a polar grid, and *vice versa*.

NUFFTs offer a precise and efficient method for computing equation (5). This method involves first computing the Fourier coefficients on a polar grid using a sequence of one-dimensional FFTs along radial lines. Then, the computed coefficients are convolved with a compactly supported spreading kernel φ, and this convolution is evaluated on a uniform grid (see Fig. 2 ▸). Subsequently, an inverse Fourier transformation is performed on the convolution values on the uniform grid, followed by division by the Fourier transform 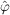 of the kernel, *i.e.*


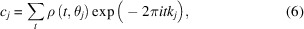




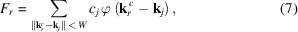




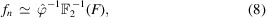

where 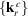 is a Cartesian grid, 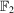 is the 2D Fourier transform, equations (6) and (8) are computed via FFTs, and *W* is the spreading width of the convolution in equation (7). For an appropriately chosen kernel, the NUFFT has an error of ε if *W* is chosen to span approximately *w* = 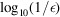 grid points per dimension. The computational complexity of equations (6)–(8) is 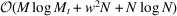 , where *M*_*t*_ is the number of points in the radial direction of the polar grid. Since 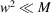 , this results in a massive speedup compared with the direct computation of equation (5). The Radon transform can similarly be computed by performing the above steps in reverse order. In this work we have used the cuFINUFFT (Shih *et al.*, 2021 ▸) library to compute NUFFTs. For a detailed discussion on the topic, we refer the reader to Dutt & Rokhlin (1993 ▸), Fessler & Sutton (2003 ▸), Greengard & Lee (2004 ▸), Barnett *et al.* (2019 ▸) and Barnett (2021 ▸).

## 3. Model-based iterative reconstruction

An alternate approach to FBP methods is to rely on iterative methods, such as MBIR. Although these methods have longer processing times, they produce better quality reconstructions when compared with FBP methods. This is especially noticeable when a smaller number of projections are available. This is because iterative methods are able to incorporate *a priori* information as a constraint on the optimization process. We refer the reader to ASTM (2019 ▸) for a more detailed discussion. Iterative methods seek a solution *f* by minimizing the difference between its Radon transform and projection data *b*, *i.e.*


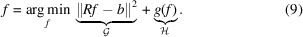

Here we set up the objective function as a least-squared problem. The target is to iteratively search for *f* that minimizes the ℓ^2^-norm of difference between *Rf* and *b* while penalizing violation of the constraint by *g*. Now we differentiate equation (9) with respect to *f* and equate the result to 0. The gradient of the first term is 

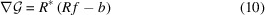

where 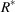 is the adjoint of equation (2), 

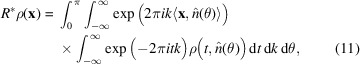

which is simply equation (4) without the scaling |*k*|. The operators *R* and 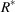 can be efficiently computed using NUFFT.

In the results presented here, we choose 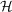 to be a total variation penalty in equation (9). We follow the mathematical approach presented by Venkatakrishnan *et al.* (2013 ▸) and Mohan *et al.* (2014 ▸) to model 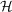 as a *q*-generalized Gaussian Markov random field (qGGMRF), 

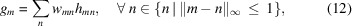




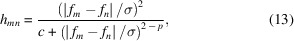

where *h* is defined over a 1-hop neighborhood of *m*, with *m* and *n* being integer coordinates on the three-dimensional uniform grid. The weights *w*_*mn*_ are the Gaussian weights that partition the unity and are inversely proportional to the distance between *m* and *n*. Hyper-parameters *c*, *p* and σ are used to control the strength of the penalty term. The term 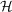 is an algebraic expression, and can easily be differentiated.

In this work, we have used a monotonic accelerated gradient method with restart detailed by Giselsson & Boyd (2014 ▸), but it is possible to use other optimizers.

## 4. Implementation

When it comes to implementing software solutions, performance is a critical factor. In this section we discuss some of the important implementation details that have a significant impact on the performance of *tomoCAM*. These include factors such as memory management and hiding PCIe latency efficient GPU caching. To achieve both high performance and user-friendliness, we utilize a blend of C++, CUDA and Python. The data structures of *tomoCAM* are implemented in C++, while most of the mathematical functions are coded using CUDA. To efficiently handle large datasets, a two-tier partition scheme is employed to seamlessly stream data into and out of GPU memory. To address the vast number of pixels in a typical synchrotron micro- or nano-CT sinogram, which can exceed 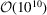 , we have carefully optimized the memory usage in the implementation of *tomoCAM*. For instance, to minimize the memory footprint, we pass large arrays that contain frequently accessed data such as the most recent solution, projection data and gradient as references rather than copies, which is the default behavior in C++. We avoid allocating two large arrays by updating the values in-place. This reduces the number of large allocations to six, saving 25% RAM. We have implemented various strategies to minimize the memory footprint, including the following:

(i) Quantities are never stored as complex numbers in the host memory. This additionally helps with the amount of data copied to and from the GPU memory.

(ii) Instead of duplicating data, partitions contain pointers to memory locations in the parent array.

(iii) Gradients are updated in place when computing the total variation constraint.

(iv) Projection data are reordered into sinogram form for fast contiguous readouts.

### 4.1. GPU optimizations

**Figure 3:**
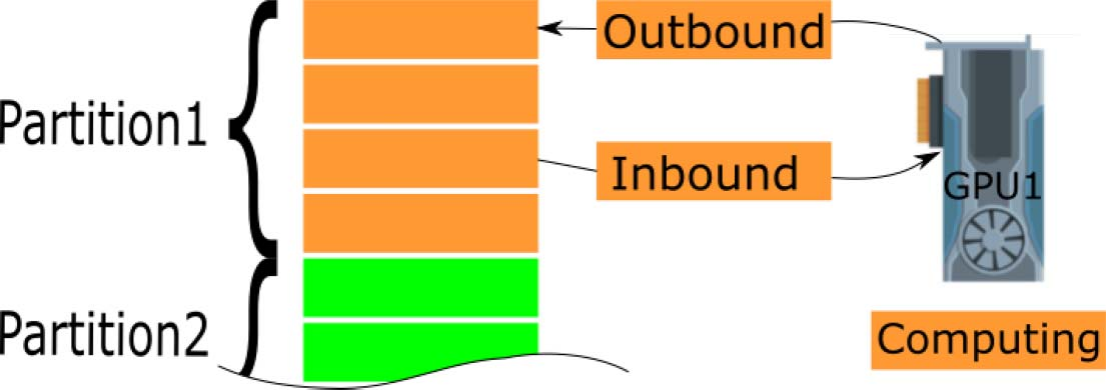
Large arrays are partitioned along the axis of rotation using a two-tier partitioning scheme. Larger, tier-1, partitions are per GPU. Smaller, tier-2, partitions (orange and green rectangles) are asynchronously streamed into GPU to overlap data transfers and computations.

While GPUs are highly efficient in performing complex calculations, the latency over the PCIe bus remains a significant bottleneck for GPU-accelerated software implementations. To minimize runtime and maximize throughput from CPU to GPU memory, we employ a combination of techniques. These include asynchronous transfers, OpenMP threads and a two-tier data partitioning scheme. The partitioning is done along the axis of rotation, with the data first divided into as many partitions as there are available GPU devices. Each partition is then further subdivided into smaller chunks, with the optimal size depending on the GPU device’s available memory. The sub-partitions are streamed to GPU memory, and to minimize the memory footprint they do not create deep copies of the data. Fig. 3 ▸ provides an overview of this process. By utilizing these techniques, we can significantly reduce the impact of the PCIe bottleneck and achieve higher performance in our GPU-accelerated software implementations. Some of the other optimizations and features of *tomoCAM* include the following:

(i) Since the axis of rotation may not be aligned with the center of the image, we use the Fourier shift property to efficiently move the rotation axis to the center of the image.

(ii) We use OpenMP threads to parallelly launch level-1 partitions on all the available GPUs, as well as to stream data into GPU memory.

(iii) To improve the cache efficiency, we utilize GPUs’ 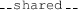 memory to store data that are accessed multiple times, such as when computing the ‘total variation’ constraint.

(iv) A Python front-end and *NumPy* compatibility are provided via the *pybind11* project (Jakob *et al.*, 2017 ▸).

## 5. Numerical experiments

We tested *tomoCAM* with publicly available phantoms and measured datasets. Here, we present a comparison of reconstructed results using *tomoCAM*, *SVMBIR*, *TIGRE* (Biguri *et al.*, 2016 ▸, 2020 ▸) and FBP using gridrec available in the *Tomopy* package (Gürsoy *et al.*, 2014 ▸). Each reconstruction and line profile (*B*) is scaled with *s* and shifted with Δ, where 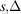 = 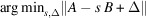 to the ground truth (*A*) before plotting. In the case of experimental data, we rescale reconstructions from *SVMBIR* and gridrec with the one obtained from *tomoCAM*. The total variation constraint used in *SVMBIR* is slightly different from the one used in *tomoCAM* [see the theory section of SVMBIR (2020 ▸)]. *SVMBIR* uses ten nearest neighbors, while *tomoCAM* uses 26 of them, to evaluate equation (12). We believe parameters can be fine-tuned for *tomoCAM* and *SVMBIR* to produce equivalent results. The primary comparison with *SVMBIR* is to demonstrate performance gains, rather than comparing two different constraints or image quality. All the tests were carried out on a single machine with (i) 2 × Intel(R) Xeon(R) CPU E5-2620 v4 @ 2.10 GHz; (ii) 4 × Tesla P100 GPUs; (iii) 128 GB RAM.

**Figure 4:**
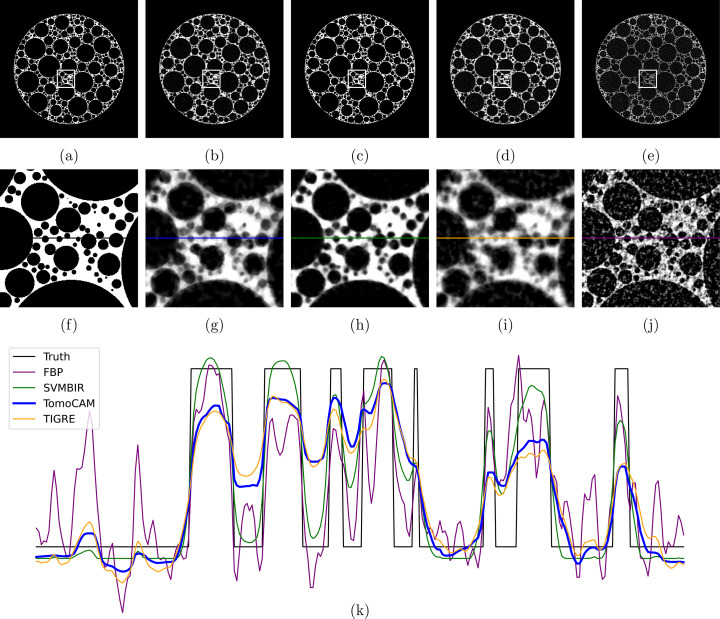
A compassion of reconstruction methods for a foam phantom with 128 projections. (*a*) Ground truth, (*b*) *tomoCAM*, (*c*) *SVMBIR*, (*d*) *TIGRE* (IRN-TV-CGLS) and (*e*) gridrec. Panels (*f*), (*g*), (*h*), (*i*) and (*j*) are the zoomed-in regions of interest represented by the boxes in (*a*), (*b*), (*c*), (*d*) and (*e*), respectively, and (*k*) displays the line profiles on (*f*), (*g*), (*h*), (*i*) and (*j*). Reconstructions using *tomoCAM*, *SVMBIR* and *TIGRE* (IRN-TV-CGLS) result in images with low noise when compared with gridrec. The relative root-mean-square error values when compared against the ground truth for gridrec, *SVMBIR*, *TIGRE* and *tomoCAM* are 0.95, 0.33, 0.53 and 0.47, respectively. Here *tomoCAM* is about 9× faster than *SVMBIR*, and 20× faster than *TIGRE* (IRN-TV-CGLS).

In the first experiment, we compare the reconstruction of a foam phantom from all three codes. A foam phantom and its projection data of size (128 × 16 × 2048) was generated using the *foam_ct_phantom* package (Pelt *et al.*, 2022 ▸). A full slice from the phantom in Fig. 4 ▸(*a*) is compared with the reconstruction obtained from each code (*SVMBIR*, *tomoCAM* and gridrec) in Figs. 4 ▸(*b*)–4(*e*). This is followed by zoomed-in regions of each image in Figs. 4 ▸(*f*)–4(*j*). A line profile from each of the zoomed-in regions is then compared in Fig. 4 ▸(*k*). It is evident from the results that both *tomoCAM* and *SVMBIR* are effective at suppressing the noise. One major advantage of *tomoCAM* is that it can obtain equivalent results in an order-of-magnitude faster time.

**Figure 5:**
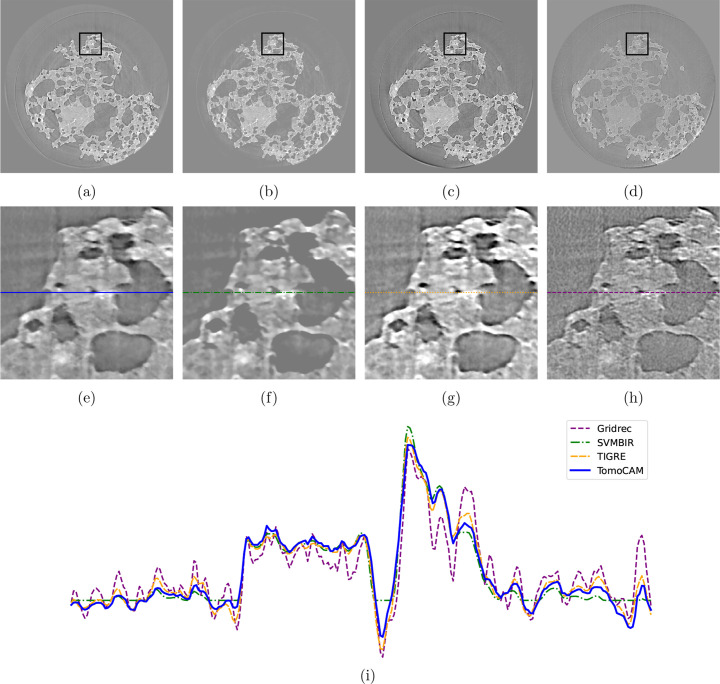
Reconstructions for Tomobank dataset ID 25, micro-CT *in situ* study of rock permeability with 400 projections, with (*a*) *tomoCAM*, (*b*) *SVMBIR*, (*c*) *TIGRE* (IRN-TV-CGLS) and (*d*) gridrec. Panels (*e*), (*f*), (*g*) and (*h*) are zoomed-in regions of interest represented by the boxes in (*a*), (*b*), (*c*) and (*d*), respectively. Panel (*i*) displays the line profiles for (*e*), (*f*), (*g*) and (*h*). A circular mask was applied to all the reconstructions. While *tomoCAM*, *SVMBIR* and *TIGRE* (IRN-TV-CGLS) do an excellent job at suppressing the noise when compared with gridrec, *tomoCAM* is approximately 15× faster than *SVMBIR* and 22× faster than *TIGRE* (IRN-TV-CGLS).

Next, we evaluate the reconstruction of two experimental datasets obtained from diverse synchrotron light sources that are accessible through Tomobank (Carlo *et al.*, 2018 ▸) (see Figs. 5 ▸ and 6 ▸). For each dataset, the available number of projections is notably lower than what is typically expected, which follows the general rule of thumb that it should be as many as the number of pixel columns in the camera sensor.

**Figure 7:**
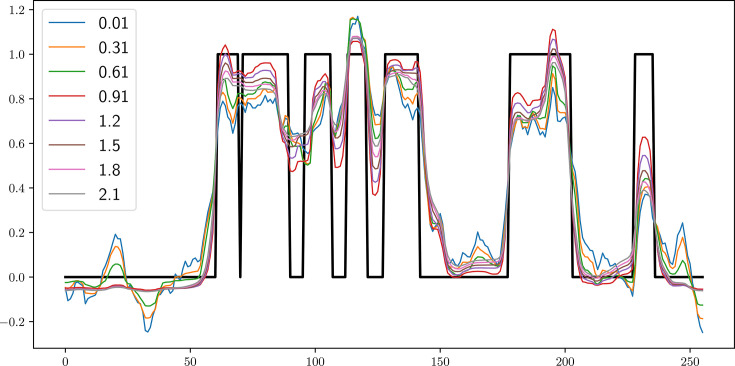
A small subset of slices were reconstructed using a range of hyper-parameters σ to determine an optimal reconstruction quality.

Using Beer–Lambert’s law (Swinehart, 1962 ▸), *b* in equation (9) is defined as 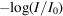 , where *I* is the measured intensity and *I*_0_ is the beam intensity without the sample blocking the view. The selection of hyper-parameters can influence the quality of reconstruction. However, in practice *p* = 1.2 (Mohan *et al.*, 2014 ▸) and *c* = 0.0001 have shown good performance across multiple datasets. The strength of the qGGMRF constraint is controlled by the parameter σ. Lower values of σ increase the contribution of the constraint, resulting in smoother profiles, while higher values decrease the contribution. We evaluate a spectrum of σ values on a small subset of dataset, in order to ascertain the hyper-parameter value that yields a desirable quality of data reconstruction, see Fig. 7 ▸. Without a quantitative measure of reconstruction quality, we rely on a human expert to determine the fitness of the reconstruction. In order to automate this process, we are currently working on developing new machine-learning approaches to recommend optimal hyper-parameters based on the features of the data.

**Figure 8:**
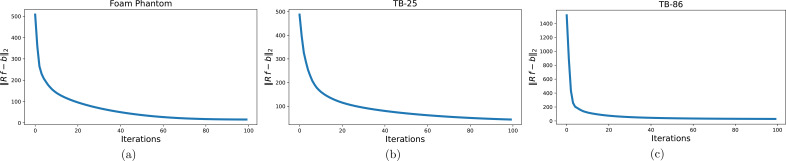
L2 error versus iterations for (*a*) a foam phantom, (*b*) TB-25 and (*c*) TB-86. Although the optimizer converges quickly, the resolution of smaller features may need extra iterations.

For gridrec we chose the Butterworth filter with order 2, and the cutoff frequency was set to 0.25, which is typical for a synchrotron tomographic reconstruction. For *SVMBIR* we choose hyper-parameters as *T* = 1 and σ_*x*_ = {0.98, 2.1, 1.1} × 10^−4^ for the phantom, Tomobank dataset id 25 (TB-25) and Tomobank dataset id 86 (TB-86), respectively, in order to produce similar quality reconstructions as *tomoCAM*. Fig. 8 ▸ shows the convergence rates of the foam phantom, TB-25 and TB-86 samples.

We follow a similar pattern to Fig. 4 ▸ for plotting images and line profiles. The first row of images depicts full slices, followed by zoomed-in regions, and then a line profile is taken from the middle of each zoomed-in region. Given their mathematical similarity, we expect that conducting a thorough hyper-parameter search would yield comparable outcomes from both *tomoCAM* and *SVMBIR*. Table 1 ▸ shows a comparison of the time taken by each code. Small discrepancies between *tomoCAM* and *SVMBIR* are due to the difference in hyper-parameters, as well as how each of the packages implements the qGGMRF constraint. We use equation (12) directly, whereas *SVMBIR* uses a surrogate function to approximate it. We would also like to note that *TIGRE* uses a fundamentally different approach to enforce the total variation constraint (Biguri *et al.*, 2016 ▸). We have used the default hyper-parameters for reconstructions with *TIGRE* (IRN-TV-CGLS). We assume that a systematic search for hyper-parameters will produce similar quality results as *tomoCAM* and *SVMBIR*.

**Figure 9:**
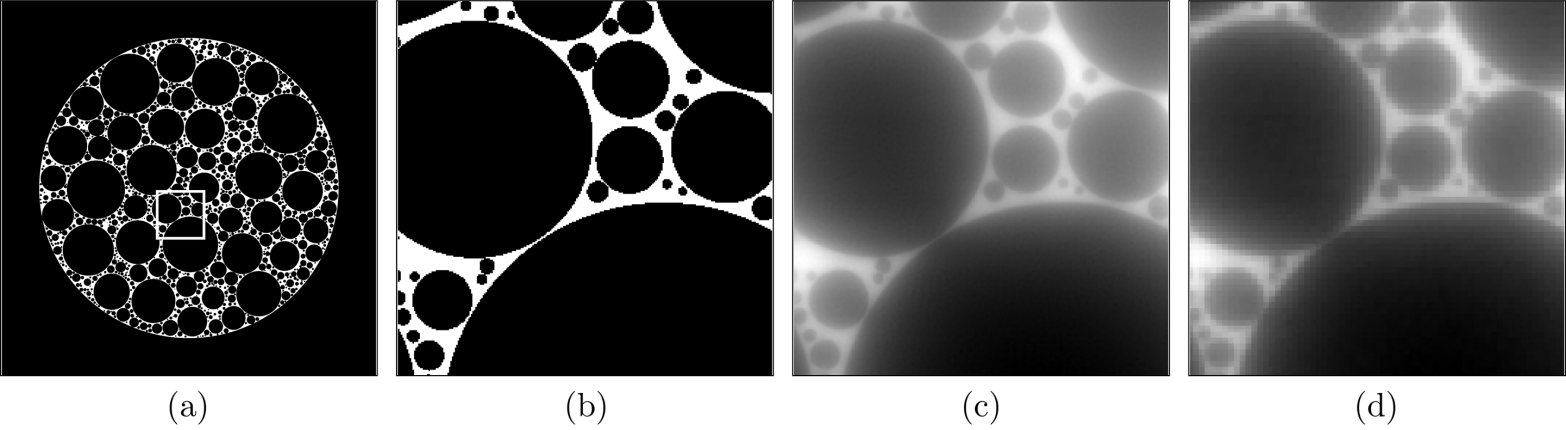
Hierarchical method. The optimization process begins with lower-resolution projection data. The result from this lower-resolution reconstruction is then up-sampled and serves as the initial point for the next cycle, which needs fewer iterations. Panel (*a*) represents the ground truth, and panel (*b*) is an area of interest within (*a*). Panel (*c*) is the same region of interest from a 100-iteration reconstruction at full resolution, and panel (*d*) shows the reconstruction achieved through the hierarchical method. The hierarchical method takes one-fifth of the time required for reconstruction at the full resolution to achieve the same convergence.

We have also implemented a hierarchical reconstruction method that iteratively refines the solution by starting at a low resolution and gradually increasing the resolution. This reduces the number of optimization iterations required at higher resolution, leading to significant speedups. In the example shown in Fig. 9 ▸, for a foam phantom with 512 projections of 512  ×  2048 resolution, we apply this hierarchical approach. The final reconstruction resolution is (512 × 2048 × 2048). We started with projection images down-sampled to one-quarter of the original resolution, running for 60 iterations. This was followed by two cycles of up-sampling the solution, with reconstruction at the half resolution running for 30 iterations; and finally 15 iterations at the full resolution. The hierarchical method is five times faster than running at full resolution, *i.e.* 809 versus 4090 s for similar convergence values. We are working on making further improvements to the hierarchical method before it is made publicly available.

## 6. Conclusions

In this work, we have presented *tomoCAM*, a new GPU-accelerated software for reconstructing high-quality tomographic images. *tomoCAM* is capable of running model-based iterative reconstructions for large datasets with relatively modest hardware requirements, within a reasonable time. The resulting reconstructed images have lower noise when compared with the prevalent FBP methods, while being an order of magnitude faster than CPU-only MBIR implementations.

A Python-based front-end has been created for *tomoCAM*, which is specifically designed to receive *NumPy* arrays as both input and output for reconstructions. This facilitates seamless integration of *tomoCAM* into the existing workflows of beamline scientists. Although the use of MBIR is particularly advantageous in cases where there is a scarcity of available projection data, the current implementation of MBIR is quite time-consuming. Consequently, this is the primary reason why beamline scientists do not utilize MBIR even when it is advantageous. *tomoCAM* overcomes this problem, thus making MBIR reconstruction more practical, by the following:

(i) Improving efficiency: the run time has been reduced by an order of magnitude, making it faster than previous MBIR versions.

(ii) Reducing hardware requirements: it can run on machines as small as an individual desktop with a GPU, making it more accessible.

(iii) Simplifying hyper-parameter search: *tomoCAM*’s speed makes it easier to search for hyper-parameters, allowing for faster and more efficient experimentation.

(iv) Enhancing compatibility: the implementation provides a Python interface, which makes it easy to integrate with existing workflows that use FBP.

## Figures and Tables

**Figure 6:**
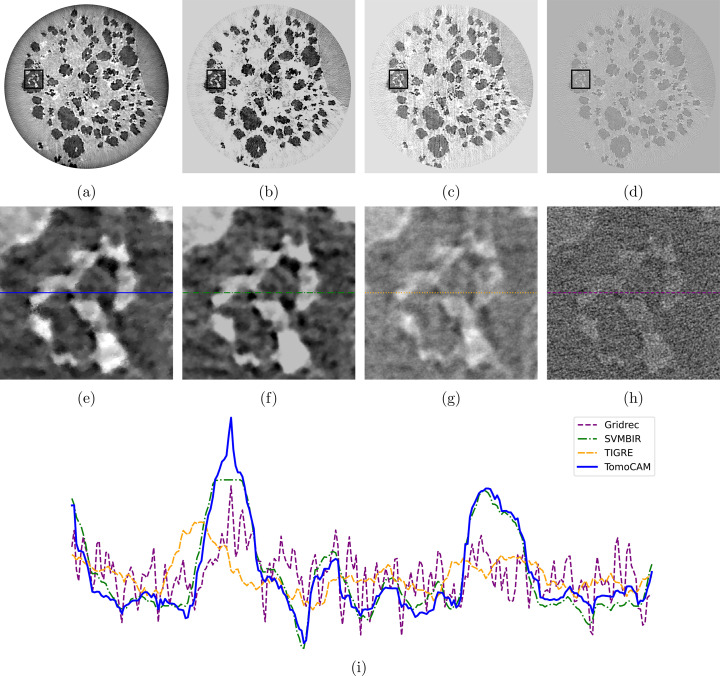
Reconstructions for Tomobank dataset ID 86, nano-CT data with sparse projection angles using 202 projections, with (*a*) *tomoCAM*, (*b*) *SVMBIR*, (*c*) *TIGRE* (IRN-TV-CGLS) and (*d*) gridrec. Panels (*e*), (*f*), (*g*) and (*h*) are zoomed-in regions of interest represented by the boxes in (*a*), (*b*), (*c*) and (*d*), respectively. Panel (*i*) displays the line profiles for (*d*), (*e*), (*f*) and (*h*). A circular mask was applied to all the reconstructions. While *tomoCAM* and *SVMBIR* both do an excellent job at suppressing the noise when compared with gridrec, *tomoCAM* is approximately 15× faster. Also, *tomoCAM* is 22× faster than *TIGRE* (IRN-TV-CGLS).

**Table 1:** A comparison of the time taken to reconstruct various datasets; iterative methods *tomoCAM*, *SVMBIR* and *TIGRE* were timed for 100 iterations

		Reconstruction time (s)			
Data set	Size	*tomoCAM*	*SVMBIR*	*TIGRE*	gridrec
Phantom	(128, 16, 2048)	93	810	1820	0.21
TB-25	(400, 128, 2048)	862	12730	18833	2.53
TB-86	(202, 128, 2448)	1210	14273	26147	3.73

## Footnotes

1 Although some authors prefer to distinguish filtered back-projection computed in real space from gridrec, it is common for gridrec to be considered a specific implementation of filtered back-projection in Fourier space (*e.g.* Barutcu *et al.*, 2021 ▸), as they are both based on approximating the inverse Radon transform through integration of filtered projection data over the projection angle (Kak & Slaney, 2001 ▸). In this work, we take the latter convention.
